# Efficacy and Safety of Co-Administered St. John’s Wort and *Ginkgo biloba* Extracts in Patients with Subjective Tinnitus: A Preliminary Prospective Randomized Controlled Trial

**DOI:** 10.3390/jcm12093261

**Published:** 2023-05-03

**Authors:** Hantai Kim, Jungho Ha, Hun Yi Park, Yun-Hoon Choung, Jeong Hun Jang

**Affiliations:** 1 Department of Otorhinolaryngology–Head and Neck Surgery, Konyang University College of Medicine, Daejeon 35365, Republic of Korea; 2 Department of Otolaryngology, Ajou University School of Medicine, Suwon 16499, Republic of Korea

**Keywords:** subjective tinnitus, *Hypericum*, Saint John’s wort, *Ginkgo biloba*

## Abstract

It is widely accepted that extracts of St. John’s wort (*Hypericum perforatum*) improve depressive symptoms, and tinnitus patients commonly presented with either mild depression or anxiety. We investigated whether co-administration of St. John’s wort and *Ginkgo biloba* extracts can suppress tinnitus. Participants with subjective tinnitus aged 30–70 years were randomly assigned to the experimental (co-administration of St. John’s wort and *Ginkgo biloba* extract; n = 20) or control (*Ginkgo biloba* extract only; n = 26) group for 12 weeks. Participants were blinded to the group assignments. After 12 weeks of treatment, no significant change in the minimum masking level on the tinnitogram was observed in either group. In the co-administration group, the Tinnitus Handicap Inventory (THI) score decreased from 34.7 (SD, 15.9) to 29.6 (16.0) (*p* = 0.102). However, the control group showed a significant decrease in THI score, from 30.5 (16.7) to 25.6 (17.1) (*p* = 0.046). Regarding the Short Form-36 Health Survey (SF-36), only the “Social Functioning” domain score changed significantly after extract co-administration, from 74.5 (21.5) to 83.9 (20.5) (*p* = 0.047). Co-administration of St. John’s wort and *Ginkgo biloba* extracts did not improve the symptoms of subjective tinnitus compared to administration of *Ginkgo biloba* extract alone.

## 1. Introduction

Tinnitus refers to the perception of a sound without an external source. Although some differences are evident among countries, 5–30% of people experience tinnitus once in their lifetime [1,2,3,4,5], among whom 1–7% complain of severe tinnitus [3,4,5,6]. The demand for tinnitus treatment is high but current treatment protocols are limited. A survey of audiologists and patients revealed that almost 80% considered treatment outcomes not to be successful [7]. The current tinnitus treatment standard is tinnitus retraining therapy (TRT), which is based on the neurophysiological model of Jastreboff and management of hearing loss [8,9,10,11]. Medications are not very effective, although some are valuable in patients with secondary tinnitus caused by specific diseases. Clinical guidelines recommended that even routine medications should be avoided [10,11].

Tinnitus treatment can be challenging, with TRT and hearing aids being the most evidence-based approaches. Comprehensive TRT can be a time-consuming process that requires patience from both patients and physicians, and the low rate of hearing aid use further compounds the issue [12,13]. As a result, many tinnitus sufferers remain dissatisfied with their treatment outcomes. A questionnaire study conducted by Husain et al. in 2018 found that 78.8% of respondents reported dissatisfaction with their tinnitus treatment. They even reported medication emerging as the preferred treatment option over counseling or hearing aids [7]. While clinical guidelines discourage the routine use of medications, prescriptions for medications to treat tinnitus are still frequently required [3]. Therefore, up until now, ongoing research efforts have continued in the search for an effective medication to alleviate tinnitus symptoms.

Several studies have aimed to identify effective medications [14]. These studies can be divided into two categories: one focuses on the use of commonly prescribed psychiatric medications, such as anxiolytics and antidepressants. As many tinnitus patients complain of depressive symptoms, the effectiveness of antidepressants has been explored [15,16,17,18,19,20,21]. Although some studies reported positive outcomes, the data are controversial. In addition, given concerns about side effects, such drugs are prescribed for only patients with symptoms of either depression or anxiety [11,22]. On the other hand, there have been endeavors to discover novel compounds, particularly those sourced from natural products, in order to investigate their potential efficacy against tinnitus. One such well-known substance is *Ginkgo biloba*.

*Ginkgo biloba* extract, a natural product-derived material, has been widely used in tinnitus patients, although there is some disagreement in the literature regarding its effectiveness [23]. While one systemic review found it to be effective, another did not [24,25]. Despite this, it has become popular due to its ability to provide some relief to tinnitus sufferers without the burden of the side effects associated with antidepressants or anxiolytics. However, *Ginkgo biloba* extract cannot fully meet the medical needs of tinnitus patients, and there is continued interest in exploring other natural materials. One such agent that has garnered attention is St. John’s wort. Extracts of St. John’s wort (*Hypericum perforatum*) (an herb) improve depressive symptoms [26,27]; the incidence of side effects is lower than for conventional antidepressants [28,29]. However, the extracts are not more effective than non-biological antidepressants and are thus mainly prescribed to treat dysthymia rather than major depressive disorder (MMD) [30]. Tinnitus patients often exhibit mild depression or anxiety. We hypothesized that St. John’s wort might aid such patients, and thus conducted this preliminary randomized controlled trial to determine whether co-administration of St. John’s wort and *Ginkgo biloba* extracts suppresses tinnitus. The safety of the combination was also evaluated.

## 2. Materials and Methods

### 2.1. Participants

**Figure 1:**
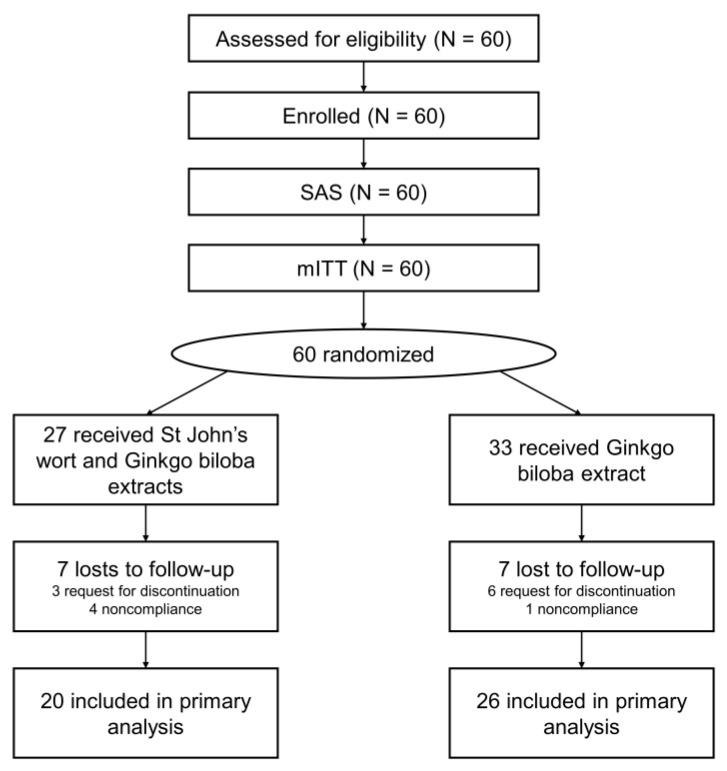
CONSORT flow chart. SAS, safety analysis set; mITT, modified intention-to-treat; PP, per protocol.

Participants with subjective tinnitus aged between 30 and 70 years were recruited from March 2019 to May 2020 from among those who visited the Department of Otolaryngology, Ajou University Hospital (Suwon, Republic of Korea). We recruited 60 participants and assigned 27 to the experimental group (St. John’s wort and *Ginkgo biloba* extracts) and 33 to the control group (*Ginkgo biloba* extract only). However, 7 patients in each group were lost to follow-up within 12 weeks. Thus 46 participants were included in the final analysis (Figure 1).

The inclusion criteria were as follows: subjective tinnitus for ≥3 months; no pre-scription of any antipsychotic, anxiolytic, antidepressant, or other medication to treat a neuropsychiatric disorder; no history of a neuropsychiatric disorder; and consent to visit us during the study period. The exclusion criteria were aged <30 or >70 years; a mean pure-tone threshold at 500–4000 Hz > 25 dB HL; pulsatile tinnitus attributable to an anatomical lesion; current treatment for severe cardiac, pulmonary, hepatic, or renal disease; a cardiac problem within 4 weeks before the study; abnormal baseline laboratory data; and an allergy to St. John’s wort extract or *Ginkgo biloba*.

### 2.2. Assessments

Before and after the administration of the treatment, participants underwent a total of six tests, including pure-tone audiometry (PTA), tinnitogram, and completion of four questionnaires: the Tinnitus Handicap Inventory (THI), the Beck Depression Inventory (BDI), the Pittsburgh Sleep Quality Index (PSQI), and the Short Form-36 Health Survey (SF-36).

The PTA test assessed pure tone thresholds at frequencies of 0.5, 1, 2, and 4 kHz, and the average of these values was used as a screening tool. Participants with an average pure tone threshold exceeding 25 dB were not included in the study. If individuals with hearing loss were included in the study, there would be the potential for bias to be introduced due to the variability in the magnitude of hearing loss. Given the small sample of the study (30 participants), it was considered that it would be difficult to effectively control for the impact of hearing loss on tinnitus. Therefore, the decision was made to exclude individuals with hearing loss from the study.

A tinnitogram is a test that aims to identify the specific features of tinnitus experienced by a participant. The test involves “pitch matching”, which consists of presenting a sound with a frequency range of 0.125–12 kHz, at a loudness 10 dB higher than the pure tone threshold, for 2–3 s to find the sound that most closely resembles the tinnitus heard by the participant. The stimulus sound provided initially consists of pure tones, but if the subject does not perceive it, narrow band noise or white noise may be used instead. Based on the pitch (frequency) matched, the tester increases and decreases the loudness of the sound by 1 dB to find the intensity of the stimulus that the subject perceives as being equal to the loudness of their tinnitus. The magnitude of the sound obtained is called “loudness” and is expressed in dB SL. Using the loudness level, the test gradually increases the volume of the sound to find the minimum volume at which the tinnitus heard in the ear is masked. This minimum volume is called the “minimal masking level” and is expressed in dB HL. The loudness and minimal masking level were evaluated in both ears before and after the administration of the treatment. It is important to note that even if a participant reports subjective tinnitus, the tinnitogram does not always confirm the specific features of the tinnitus as perceived by the subject. Therefore, we chose the change in THI score from pre- to post-treatment as our primary outcome rather than the values obtained from the tinnitogram.

The THI is a commonly used questionnaire to evaluate the impact of tinnitus on an individual’s quality of life [31]. The THI consists of 25 questions that are scored as “no” (0 points), “sometimes” (2 points), or “yes” (4 points). The total score ranges from 0 to 100, with higher scores indicating a greater level of distress. The questionnaire is divided into 3 subscales: functional (11 questions), emotional (9 questions), and catastrophic (5 questions). The total score is used to classify the severity of tinnitus, with scores of ≤16 indicating slight, 18–36 indicating mild, 38–56 indicating moderate, 58–76 indicating severe, and 78–100 indicating catastrophic tinnitus.

The BDI is a questionnaire used to assess symptoms of depression in individuals, and we included it in our study because St. John’s wort has been shown to be effective in reducing depressive symptoms [32]. The questionnaire contains 21 items, and participants select the response that best represents their current feelings, with scores ranging from 0 to 3 for each question. The total score ranges from 0 to 63 points, with a score of 9 or lower indicating no depression, 10–15 indicating mild depression, 16–23 indicating moderate depression, and 24 or higher indicating severe depression.

As sleep disturbance is frequently reported among individuals with tinnitus, we included a questionnaire to assess the quality of sleep [33]. The PSQI is a commonly used tool for this purpose. It comprises 7 items related to sleep, totaling 19 questions, and the scores range from 0 to 21. A total score of 5 or lower indicates good sleep quality, while a score greater than 5 suggests poor sleep quality.

The SF-36 questionnaire was given to evaluate the subject’s general quality of life [34]. There are 36 responses in total across 11 domains, which are divided into 8 categories to describe the subject’s current quality of life. These categories include “Physical Functioning (PF)”, “Role-Physical (RP)”, “Bodily Pain (BP)”, “General Health (GH)”, “Vitality (VT)”, “Social Functioning (SF)”, “Role-Emotional (RE)”, and “Mental Health (MH)”.

### 2.3. Randomization

To ensure that participants were randomly assigned to either the experimental (St. John’s wort extract and *Ginkgo biloba*) or control group (*Ginkgo biloba*), we made a random assignment list using a computer program that generated 100 random numbers. Each number was 0 for the control group or 1 for the experimental group. When a participant was recruited, they were assigned to the experimental group or the control group according to the pre-made random assignment list. The random assignment list was kept confidential and was only accessed by the study coordinator to ensure the integrity of the randomization process. No stratification or blocking was used in the randomization process.

### 2.4. Intervention

We enrolled patients with subjective tinnitus ≥ 3 months in duration with mean pure-tone thresholds 500–4000 Hz at ≤25 dB HL. In the experimental group, St. John’s wort extract (Noiromin; Yuyu Pharma Inc., Seoul, Republic of Korea) and *Ginkgo biloba* extract EGb 761^®^ (Tanamin; Yuyu Pharma Inc., Seoul, Korea) were co-administered twice daily in doses of 300 mg (total = 600 mg) and 80 mg (total = 160 mg), respectively. The control group only received *Ginkgo biloba* extract EGb 761^®^ (two 80-mg doses per day). The duration of medication was 12 weeks.

To prevent any potential bias that may occur if participants were aware of which medication they were receiving, the study drugs were produced to have the same appearance. This was achieved by carefully matching the color, shape, and size of the drugs, as well as any packaging or labelling. To ensure proper administration, the study coordinator was responsible for dispensing the medications to participants, ensuring that each participant received the correct drug as per the random assignment list.

Throughout the study, participants made three visits to our clinic, during which they received counseling on how to habituate to negative tinnitus reactions. The counseling focused on the role of environmental sound and provided information on the physiology of the auditory system and sound pathway, based on the neurophysiological model of Jastreboff [8]. The counseling aimed to help participants better understand their tinnitus and learn how to cope with it effectively, regardless of which medication they were receiving.

### 2.5. Outcomes

We analyzed both subjective and objective changes in tinnitus symptoms. Questionnaire scores were compared before and after treatment. Changes in tinnitus severity were evaluated using the THI. We used the BDI, PSQI, and SF-36 scores to assess changes in depressive symptoms, sleep quality, and quality of life, respectively. Objective tinnitus changes (loudness and minimum masking level) were assessed by tinnitogram before and after treatment.

### 2.6. Statistical Analyses

The statistical analyses in this study involved a variety of tests and methods to compare and analyze the data. For continuous variables, Student’s t-test was used to compare the means between two groups. For categorical variables, the chi-squared test was used to compare the proportions between groups. The Wilcoxon signed-rank test was also used to compare differences before and after treatment, specifically to assess changes in the Tinnitus Loudness and Minimal Masking Levels in the tinnitogram, THI, BDI, PSQI, and SF-36 scores over time.

In addition to these univariate tests, multiple linear regression was performed to identify factors that may affect the post-treatment THI scores. This multivariate analysis allowed for the examination of multiple variables simultaneously to determine their individual and combined effects on the outcome variable of interest.

All statistical analyses were performed using IBM SPSS Statistics for Windows (ver. 23.0; IBM Corp., Armonk, NY, USA). A significance level of *p* < 0.05 was used to determine statistical significance. Overall, the statistical analyses in this study were comprehensive and utilized a variety of methods to explore the relationships between variables and to determine the factors that may be associated with changes in tinnitus severity.

## 3. Results

### 3.1. Characteristics of the Groups before Drug Administration

Forty-six participants completed all follow-up visits; their characteristics are shown in Table 1. There was no group difference in age, sex, hearing level, or questionnaire scores. Tinnitus severity was determined by the THI as follows: ≤16, slight; 18–36, mild; 38–56, moderate; 58–76, severe; and 78–100, catastrophic [31]. In the experimental group, 3 (15.0%), 9 (45.0%), 6 (30.0%), and 2 (10.0%) patients had slight, mild, moderate, and severe tinnitus, respectively. In the control group, 6 (23.1%), 10 (38.5%), 8 (30.8%), and 2 (7.7%) patients had slight, mild, moderate, and severe tinnitus (*p* = 0.904), respectively.

### 3.2. Changes in the Tinnitogram before and after Extract Administration

**Figure 2:**
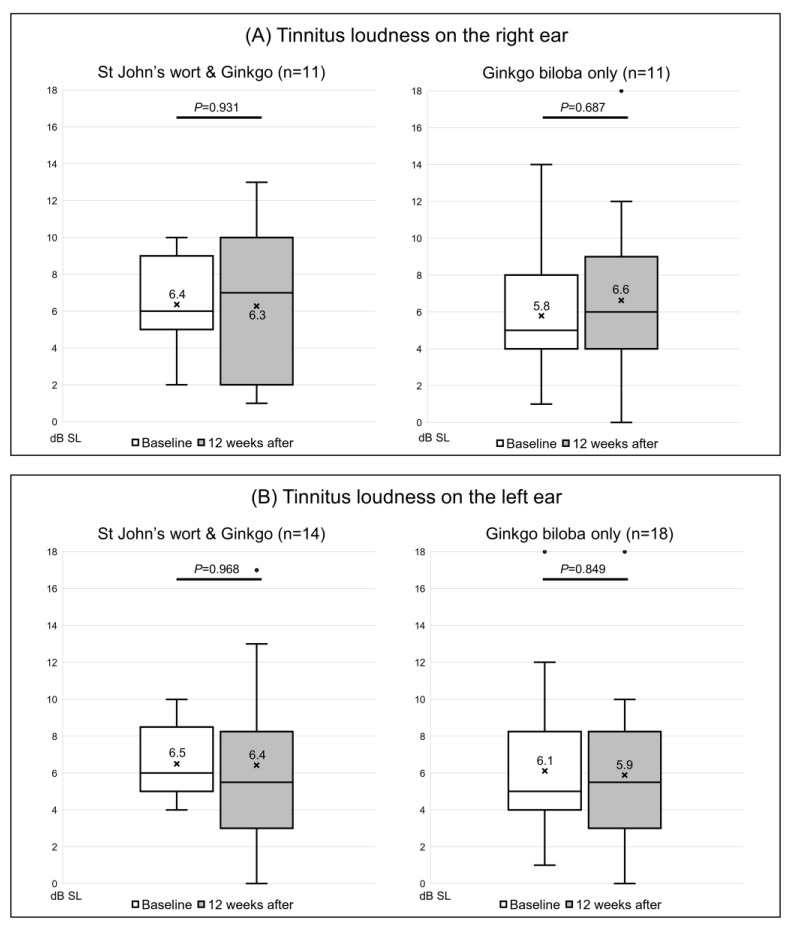
Changes in the Tinnitus Loudness (dB SL) on the tinnitogram. (**A**) The loudness on the right ear and (**B**) on the left ear. The box-and-whiskers plot show the median, upper lower quartile (boxes), and minimum and maximum (excluding outliers) values, as well as outliers (1.5-fold the upper/lower quartile values). X = mean value. The Wilcoxon signed-rank test was used to determine statistical significance.

**Figure 3:**
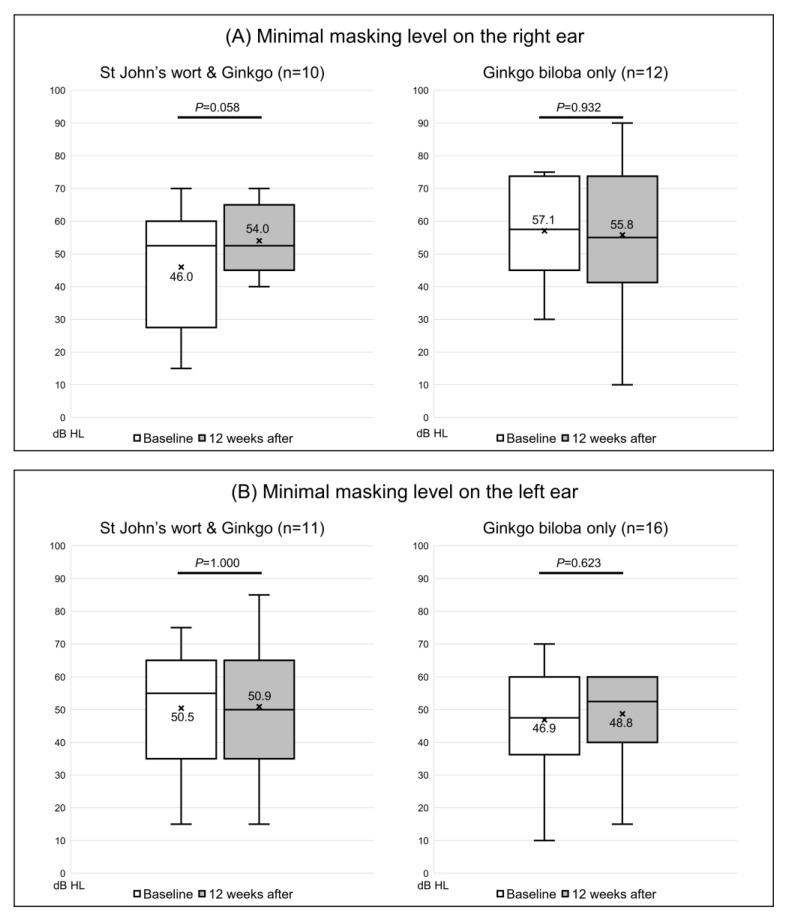
Changes in the Minimal Masking Level (dB HL) on the tinnitogram in 22 right ears and 27 left ears. (**A**) The masking level on the right ear and (**B**) on the left ear. The box-and-whiskers plot show the median, upper lower quartile (boxes), and minimum and maximum (excluding outliers) values, as well as outliers (1.5-fold the upper/lower quartile values). X = mean value. The Wilcoxon signed-rank test was used to determine statistical significance.

Of the 20 participants given both St. John’s wort and *Ginkgo biloba*, the tinnitus loudness was not matched in 5, while 4 were matched only in the right ear and 5 only in the left ear; the loudness in both ears was matched for the other 6 patients. In the right ear, the loudness was 6.4 ± 2.7 dB SL before extract administration and was almost unchanged (6.3 ± 3.9 dB SL) after treatment (*p* = 0.931) (Figure 2A). In the left ear, the loudness changed from 6.5 ± 2.2 dB SL to 6.4 ± 4.7 dB SL (*p* = 0.968) (Figure 2B). Of the 26 control subjects, the loudness was not matched in 7, 3 were matched only in the right ear and 7 only in the left ear, while 9 were matched in both ears. Loudness increased from 5.8 ± 3.4 to 6.6 ± 4.2 dB SL in the right ear (*p* = 0.687) (Figure 2A), and from 6.1 ± 4.1 to 5.9 ± 4.2 dB SL in the left ear (*p* = 0.849) (Figure 2B); none of the changes were significant. The minimum masking level was matched in 22 right and 27 left ears. In the extract co-administration group, the minimum masking level increased from 46.0 ± 18.4 to 54.0 ± 10.5 dB HL in the right ear (Figure 3A). In the left ear, the change was from 50.5 ± 19.3 to 50.9 ± 19.6 dB HL; neither change was significant (*p* = 0.058 and 1.000, respectively). In the control group, the minimum masking level decreased from 57.1 ± 15.3 to 55.8 ± 22.2 dB HL in the right ear (Figure 3A), and from 46.9 ± 15.5 to 48.8 ± 12.8 dB HL in the left ear (Figure 3B); neither change was significant (*p* = 0.932 and *p* = 0.623, respectively).

### 3.3. The Tinnitus Handicap Inventory (THI) Questionnaire Responses before and after Drug Administration

**Figure 4:**
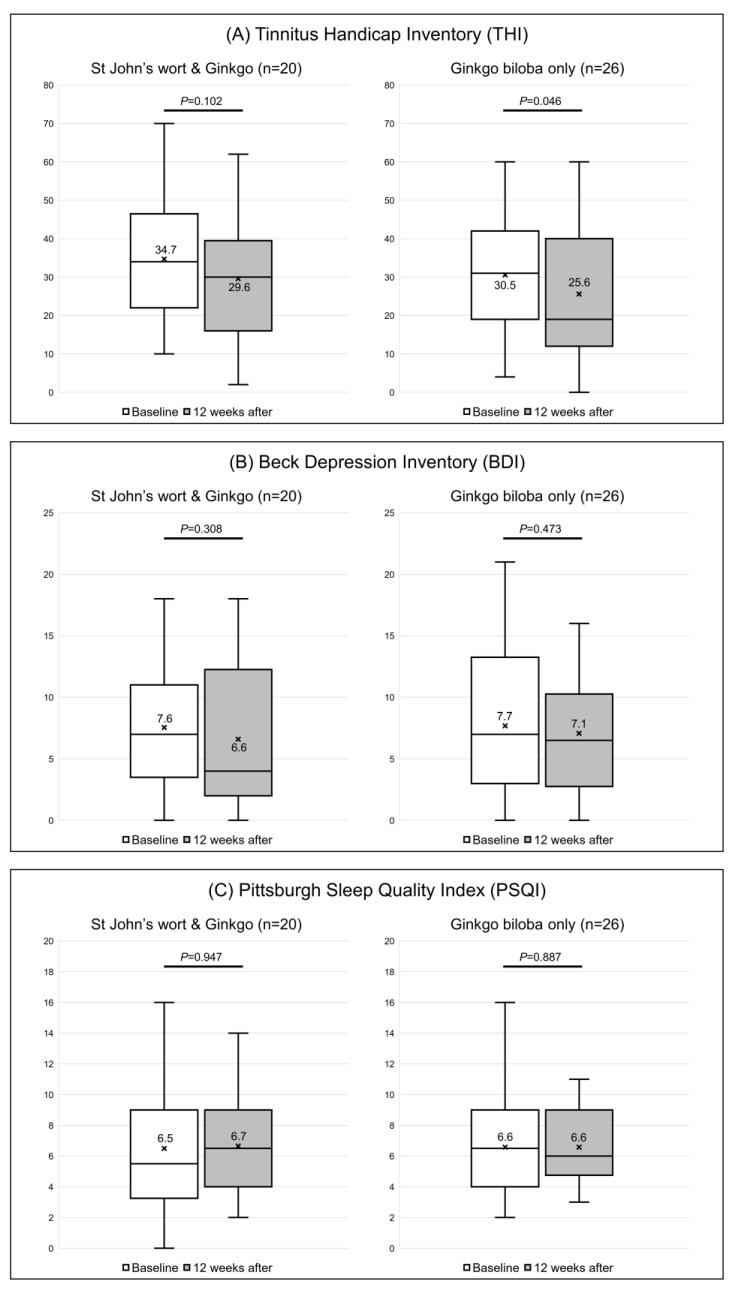
Responses to the (**A**) Tinnitus Handicap Inventory (THI), (**B**) Beck Depression Inventory (BDI), and (**C**) Pittsburgh Sleep Quality Index questionnaires of the two groups. The box-and-whiskers plot show the median, upper lower quartile (boxes), and minimum and maximum (excluding outliers) values, as well as outliers (1.5-fold the upper/lower quartile values). X = mean value. The Wilcoxon signed-rank test was used to determine statistical significance.

In participants receiving both St. John’s wort and *Ginkgo biloba* extracts, the THI score decreased from 34.7 ± 15.9 to 29.6 ± 16.0, but the difference was not significant (*p* = 0.102). Surprisingly, the change was significant in the control group; the score decreased from 30.5 ± 16.7 to 25.6 ± 17.1 (*p* = 0.046) (Figure 4A and Table 2). 

### 3.4. The Beck Depression Inventory (BDI) and the Pittsburgh Sleep Quality Index (PSQI)

The BDI score decreased from 7.6 ± 4.7 to 6.6 ± 6.0 in the experimental group (*p* = 0.308) and from 7.7 ± 5.9 to 7.1 ± 5.3 in the control group (*p* = 0.473) (Figure 4B). The PSQI scores also showed no significant change. In participants who received both extracts, the PSQI score decreased from 6.5 ± 4.0 to 6.7 ± 3.3 (*p* = 0.947), and from 6.6 ± 3.4 to 6.6 ± 2.6 in those who took *Ginkgo biloba* only (*p* = 0.887) (Figure 4C).

### 3.5. Changes of the Quality of Life Using the Short Form-36 Health Survey (SF-36)

The SF-36 scores before and after extract administration were similar for most domains. Only the score for the “Social Functioning” domain changed significantly when St. John’s wort and *Ginkgo biloba* extracts were co-administered, from 74.5 ± 21.5 to 83.9 ± 20.5 (*p* = 0.047). In the co-administration group, increases were apparent in five of the eight domains, compared with two domains in the control group (Table 3).

### 3.6. Multivariate Analysis of Factors Affecting the THI Score

We used multiple linear regression to identify variables that independently affected the THI score after drug administration; only the THI score prior to drug administration had a significant effect (B = 0.752; 95% confidence interval 0.545 to 0.960; *p* < 0.001). St. John’s wort extract did not affect the THI score (0.868; –6.001 to 7.737; *p* = 0.800).

### 3.7. Adverse Events

Five participants reported adverse effects (AEs) after extract administration. In the experimental group, two participants complained of headache; one reported an eye floater that spontaneously resolved and another experienced mild dyspepsia. In the control group, one participant felt dizzy for a few minutes. All AEs were mild and resolved spontaneously.

## 4. Discussion

Tinnitus patients have a significant need for medication to manage their symptoms, as evidenced by some studies [3,7]. In addition to antidepressants and *Ginkgo biloba* extract, other medications have been investigated for their potential to improve tinnitus. Along with depression, anxiety is also a common condition experienced by tinnitus patients. Therefore, many studies have investigated drugs that may be able to reduce the symptoms of tinnitus. Benzodiazepines that bind to the neurotransmitter γ-aminobutyric acid (GABA) receptor relieve anxiety and depression [35]. Several studies have reported positive results; however, benzodiazepines reduce the anxiety associated with tinnitus rather than tinnitus itself [36,37]. The use of clonazepam, which has a lower risk of AEs, is generally supported, but it also manages anxiety rather than the primary tinnitus [10,11,38].

Studies investigating the efficacy of antidepressants in suppressing tinnitus began with tricyclic agents (TCAs) [18,19]. Although Sullivan et al. found that TCAs reduced overall tinnitus disability, 21.3% of participants dropped out of the study due to anticholinergic effects [18]. Conversely, Mihail et al. suggested that the observed effect may been due to a placebo effect [19]. Currently, given the risks of anticholinergic effects associated with TCAs, their use in tinnitus suppression is limited. Selective serotonin reuptake inhibitors (SSRIs) have also been trialed; however, their use remains controversial. Sertraline showed positive outcomes in one trial, while another study using trazodone showed no significant change [21,22]. Similar to benzodiazepines, antidepressants manage depression, not the primary subjective tinnitus [10,11,23]. Anticonvulsants and N-methyl-D-aspartate (NMDA) receptor antagonists have also been investigated; however, evidence of effectiveness is lacking [39,40,41,42]. Although it is unclear whether antidepressants or anxiolytics have a direct impact on tinnitus, managing associated depression and/or anxiety remains important in tinnitus treatment, as it can ultimately help alleviate the burden of tinnitus. To better understand how these medications can be used effectively in tinnitus patients with comorbid depression and/or anxiety, future research will require well-designed clinical studies.

This is the first study to investigate the efficacy of combined administration of St. John’s wort and *Ginkgo biloba* extracts for suppressing tinnitus. No previous study has explored the utility of St. John’s wort in this context. Thus, this preliminary study was conducted to evaluate the effectiveness of the medication and estimate its effect size, which would aid in planning a more rigorous follow-up study. In addition, in this preliminary study, we also evaluated its safety. Unfortunately, the combination treatment did not significantly suppress tinnitus. However, there were no safety issues or unexpected negative effects, but the utility of St. John’s wort should be further explored in follow-up studies. As the effect of this medication, St. John’s Wort, was not large, future studies will need to set a smaller effect size and recruit a larger number of participants.

In this study, we decided to exclude individuals with hearing loss due to the impact of hearing loss on tinnitus. Hearing loss can significantly impact tinnitus, with individuals who have severe hearing loss often experiencing more severe tinnitus. However, in future studies with a large population, it may be beneficial to include individuals with hearing loss, as many tinnitus patients experience some level of hearing loss. Additionally, including individuals with depressive symptoms (i.e., higher BDI scores) could provide further insight how to use this medication effectively. Furthermore, it may have been preferable to exclude individuals with low baseline THI scores. In our additional analysis of participants with moderate tinnitus (THI ≥ 38), the magnitude of the change was greater; the score decreased from 47.4 ± 8.5 to 36.0 ± 18.4 in the control group (*p* = 0.050) and 50.0 ± 10.6 to 37.8 ± 17.5 in the experimental group (*p* = 0.051). On the other hand, in subjects with a THI ≤ 16, it was difficult to detect effects. In planning a follow-up study, it would be important to take into account the severity of tinnitus as assessed by the THI. While this preliminary study did not use THI scores as enrollment criteria, it would be advisable to consider recruiting only patients with moderate or severe tinnitus for a more focused follow-up study.

Although 60 participants were enrolled in this study, there were 14 dropouts. They agreed to participate in the study but withdrew after discussing the study with their family. Despite careful implementation of the informed consent process, the dropout rate was somewhat high and regretful. It is worth noting that clinical trials can sometimes be met with rejection in Korea, which may have contributed to the high dropout rate [43]. It was somewhat disappointing that a control group receiving no treatment was not included in the study. The inclusion of such a group would have allowed for a clearer comparison of the effect of *Ginkgo biloba* and St. John’s Wort. If a follow-up study is planned, it should include a control group without any treatment so that a better understanding of the effectiveness of the medication can be obtained.

## 5. Conclusions

This study represents the first investigation of St. John’s wort for its tinnitus suppressive effects. After 12 weeks of administration, neither group showed a reduction in the loudness or minimal masking level on the tinnitogram. Analysis of changes in the THI scores revealed no significant changes in the functional, emotional, and catastrophic domains for either group. However, the group that received *Ginkgo biloba* only demonstrated a significant reduction in the total score of THI from 30.5 to 25.6. The administration of St. John’s wort did not result in any improvement in the BDI scores, likely because the study did not include participants with depressive symptoms. Similarly, there was no significant change in sleep quality for either group. However, St. John’s wort did lead to a significant improvement in the domain that suggest social vitality of the SF-36, from 74.5 to 83.9, with no change in other domains. Multiple linear regression, which aimed to identify factors affecting post-treatment THI scores, did not show a significant effect of St. John’s wort administration. Overall, the co-administration of *Ginkgo biloba* and St. John’s wort did not demonstrate a significant effect on tinnitus suppression compared to *Ginkgo biloba* monotherapy. Nonetheless, given the preliminary nature of this study, further clinical trials are warranted to determine the potential benefits of St. John’s wort in patients with subjective tinnitus, particularly those with comorbid depression.

## Figures and Tables

**Table 1:** Demographics, hearing levels, and scores of questionnaires at baseline of study participants by treatment groups.

	St John’s Wort & *Ginkgo biloba* (n = 20)	*Ginkgo biloba* (n = 26)	*p*-Value *
Age	50.3 (9.6)	51.3 (8.9)	0.724
Gender			0.275 **
Female	4 (20.0%)	9 (34.6%)	
Male	16 (80.0%)	17 (65.4%)	
Height (cm)	168.6 (7.9)	165.6 (9.5)	0.258
Weight (kg)	69.5 (11.9)	67.2 (11.4)	0.504
Blood pressure (BP)			
Systolic BP	131.1 (13.0)	126.9 (18.3)	0.383
Diastolic BP	81.4 (9.8)	81.5 (11.4)	0.972
Pulse (bpm)	81.7 (9.0)	77.7 (9.3)	0.158
Body temperature (°C)	36.7 (0.3)	36.6 (0.2)	0.493
Pure-tone thresholds (dB) ***			
Right ear	13.0 (5.7)	15.6 (5.2)	0.109
Left ear	15.1 (7.7)	15.0 (6.0)	0.953
36-Item Short Form Survey (SF-36)			
Physical Functioning (PF)	91.5 (11.0)	85.8 (16.8)	0.234
Role-Physical (RP)	75.0 (35.4)	77.1 (32.9)	0.847
Bodily Pain (BP)	78.0 (21.4)	78.4 (23.6)	0.954
General Health (GH)	49.7 (23.9)	55.4 (21.1)	0.403
Vitality (VT)	58.2 (18.0)	52.0 (19.2)	0.300
Social Functioning (SF)	74.5 (21.5)	75.7 (18.6)	0.851
Role-Emotional (RE)	88.2 (28.8)	82.0 (34.1)	0.539
Mental Health (MH)	72.0 (12.6)	66.9 (14.9)	0.258
Beck Depression Inventory (BDI)	7.6 (4.7)	7.7 (5.9)	0.930
Pittsburgh Sleep Quality Index (PSQI)	6.5 (4.0)	6.6 (3.4)	0.944
Tinnitus Handicap Inventory (THI)	34.7 (15.9)	30.5 (16.7)	0.396

* Independent *t* test; ** Chi-squared test; *** Mean of air conductive thresholds at 0.5, 1, 2, and 4 kHz.

**Table 2:** Changes of THI scores before and after the administration according to domains.

**St John’s Wort & *Ginkgo biloba* (n = 20)**	**Baseline**	**12 Weeks after**	Difference from Baseline to 12 Weeks *	*p*-Value **
Total score	34.7 (15.9)	29.6 (16.0)	5.1 (14.7)	0.102
Functional domain	13.5 (9.4)	13.5 (7.3)	0.0 (8.0)	0.965
Emotional domain	10.8 (7.2)	9.8 (6.7)	1.0 (6.8)	0.304
Catastrophic domain	6.8 (4.6)	6.3 (4.7)	0.5 (4.9)	0.627
***Ginkgo biloba* (n = 26)**	**Baseline**	**12 Weeks after**	Difference from Baseline to 12 Weeks ^*^	*p*-Value **
Total score	30.5 (16.7)	25.6 (17.1)	4.9 (11.6)	0.046
Functional domain	12.6 (7.8)	11.0 (7.0)	1.6 (5.9)	0.166
Emotional domain	10.6 (7.2)	9.5 (7.9)	1.1 (5.2)	0.290
Catastrophic domain	6.7 (4.5)	5.2 (4.5)	1.5 (3.7)	0.056

* Positive value means the improvement; ** Wilcoxon signed-rank test.

**Table 3:** Changes of SF-36 scores before and after the administration 1–2.

**St John’s Wort & *Ginkgo biloba* (n = 20)**	**Baseline**	**12 Weeks after**	Difference from Baseline to 12 Weeks *	*p*-Value **
Physical Functioning (PF)	91.5 (11.0)	89.7 (13.2)	1.8 (8.7)	0.589
Role-Physical (RP)	75.0 (35.4)	79.4 (31.0)	–4.4 (15.9)	0.257
Bodily Pain (BP)	78.0 (21.4)	76.9 (23.9)	1.1 (24.6)	0.753
General Health (GH)	49.7 (23.9)	55.0 (19.9)	–5.3 (14.7)	0.240
Vitality (VT)	58.2 (18.0)	61.5 (16.8)	–3.2 (18.1)	0.664
Social Functioning (SF)	74.5 (21.5)	83.9 (20.5)	–9.5 (15.7)	0.047
Role-Emotional (RE)	88.2 (28.8)	94.1 (24.3)	–5.9 (17.7)	0.180
Mental Health (MH)	72.0 (12.6)	71.1 (15.1)	0.9 (10.4)	0.607
***Ginkgo biloba* (n = 26)**	**Baseline**	**12 Weeks after**	Difference from Baseline to 12 Weeks *	*p*-Value **
Physical Functioning (PF)	85.8 (16.8)	83.8 (20.3)	2.1 (9.0)	0.395
Role-Physical (RP)	77.1 (32.9)	72.9 (35.3)	4.2 (26.2)	0.431
Bodily Pain (BP)	78.4 (23.6)	68.3 (22.6)	10.2 (25.5)	0.084
General Health (GH)	55.4 (21.1)	51.7 (17.9)	3.8 (12.7)	0.178
Vitality (VT)	52.0 (19.2)	55.6 (14.2)	–3.6 (16.0)	0.294
Social Functioning (SF)	75.7 (18.6)	70.5 (16.0)	5.2 (18.2)	0.253
Role-Emotional (RE)	82.0 (34.1)	83.4 (31.1)	–1.5 (18.3)	0.461
Mental Health (MH)	66.9 (14.9)	64.5 (16.7)	2.5 (14.9)	0.625

* Negative value means the improvement; ** Wilcoxon signed-rank test.

## Data Availability

The data presented in this study are available on request from the corresponding author.

## References

[1] Kim H.J., Lee H.J., An S.Y., Sim S., Park B., Kim S.W., Lee J.S., Hong S.K., Choi H.G. (2015). Analysis of the prevalence and associated risk factors of tinnitus in adults. PLoS ONE.

[2] Shargorodsky J., Curhan G.C., Farwell W.R. (2010). Prevalence and characteristics of tinnitus among US adults. Am. J. Med..

[3] Bhatt J.M., Lin H.W., Bhattacharyya N. (2016). Prevalence, Severity, Exposures, and Treatment Patterns of Tinnitus in the United States. JAMA Otolaryngol. Head Neck Surg..

[4] Lee D.Y., Kim Y.H. (2018). Relationship Between Diet and Tinnitus: Korea National Health and Nutrition Examination Survey. Clin. Exp. Otorhinolaryngol..

[5] Park K.H., Lee S.H., Koo J.W., Park H.Y., Lee K.Y., Choi Y.S., Oh K.W., Lee A., Yang J.E., Woo S.Y. (2014). Prevalence and associated factors of tinnitus: Data from the Korean National Health and Nutrition Examination Survey 2009–2011. J. Epidemiol..

[6] Coles R.R. (1984). Epidemiology of tinnitus: (1) prevalence. J. Laryngol. Otol. Suppl..

[7] Husain F.T., Gander P.E., Jansen J.N., Shen S. (2018). Expectations for Tinnitus Treatment and Outcomes: A Survey Study of Audiologists and Patients. J. Am. Acad. Audiol..

[8] Jastreboff P.J. (2007). Tinnitus retraining therapy. Prog. Brain Res..

[9] Jastreboff P.J., Hazell J.W. (1993). A neurophysiological approach to tinnitus: Clinical implications. Br. J. Audiol..

[10] Tunkel D.E., Bauer C.A., Sun G.H., Rosenfeld R.M., Chandrasekhar S.S., Cunningham E.R., Archer S.M., Blakley B.W., Carter J.M., Granieri E.C. (2014). Clinical practice guideline: Tinnitus. Otolaryngol. Head Neck Surg..

[11] Ogawa K., Sato H., Takahashi M., Wada T., Naito Y., Kawase T., Murakami S., Hara A., Kanzaki S. (2020). Clinical practice guidelines for diagnosis and treatment of chronic tinnitus in Japan. Auris Nasus Larynx.

[12] Chien W., Lin F.R. (2012). Prevalence of hearing aid use among older adults in the United States. Arch. Intern. Med..

[13] Moon I.J., Baek S.Y., Cho Y.S. (2015). Hearing Aid Use and Associated Factors in South Korea. Medicine.

[14] Kim S.H., Kim D., Lee J.M., Lee S.K., Kang H.J., Yeo S.G. (2021). Review of Pharmacotherapy for Tinnitus. Healthcare.

[15] Bhatt J.M., Bhattacharyya N., Lin H.W. (2017). Relationships between tinnitus and the prevalence of anxiety and depression. Laryngoscope.

[16] Langguth B., Landgrebe M., Kleinjung T., Sand G.P., Hajak G. (2011). Tinnitus and depression. World J. Biol. Psychiatry.

[17] Choi J., Lee C.H., Kim S.Y. (2021). Association of Tinnitus with Depression in a Normal Hearing Population. Medicina.

[18] Sullivan M., Katon W., Russo J., Dobie R., Sakai C. (1993). A randomized trial of nortriptyline for severe chronic tinnitus. Effects on depression, disability, and tinnitus symptoms. Arch. Intern. Med..

[19] Mihail R.C., Crowley J.M., Walden B.E., Fishburne J., Reinwall J.E., Zajtchuk J.T. (1988). The tricyclic trimipramine in the treatment of subjective tinnitus. Ann. Otol. Rhinol. Laryngol..

[20] Zöger S., Svedlund J., Holgers K.M. (2006). The effects of sertraline on severe tinnitus suffering--a randomized, double-blind, placebo-controlled study. J. Clin. Psychopharmacol..

[21] Dib G.C., Kasse C.A., Alves de Andrade T., Gurgel Testa J.R., Cruz O.L. (2007). Tinnitus treatment with Trazodone. Braz. J. Otorhinolaryngol..

[22] Baldo P., Doree C., Molin P., McFerran D., Cecco S. (2012). Antidepressants for patients with tinnitus. Cochrane Database Syst. Rev..

[23] Holstein N. (2001). Ginkgo special extract EGb 761 in tinnitus therapy. An overview of results of completed clinical trials. Fortschr. Med. Orig..

[24] von Boetticher A. (2011). Ginkgo biloba extract in the treatment of tinnitus: A systematic review. Neuropsychiatr. Dis. Treat..

[25] Rejali D., Sivakumar A., Balaji N. (2004). Ginkgo biloba does not benefit patients with tinnitus: A randomized placebo-controlled double-blind trial and meta-analysis of randomized trials. Clin. Otolaryngol. Allied Sci..

[26] Apaydin E.A., Maher A.R., Shanman R., Booth M.S., Miles J.N., Sorbero M.E., Hempel S. (2016). A systematic review of St. John’s wort for major depressive disorder. Syst. Rev..

[27] Linde K., Berner M.M., Kriston L. (2008). St John’s wort for major depression. Cochrane Database Syst. Rev..

[28] Eatemadnia A., Ansari S., Abedi P., Najar S. (2019). The effect of Hypericum perforatum on postmenopausal symptoms and depression: A randomized controlled trial. Complement Ther. Med..

[29] Abdali K., Khajehei M., Tabatabaee H.R. (2010). Effect of St John’s wort on severity, frequency, and duration of hot flashes in premenopausal, perimenopausal and postmenopausal women: A randomized, double-blind, placebo-controlled study. Menopause.

[30] Canenguez Benitez J.S., Hernandez T.E., Sundararajan R., Sarwar S., Arriaga A.J., Khan A.T., Matayoshi A., Quintanilla H.A., Kochhar H., Alam M. (2022). Advantages and Disadvantages of Using St. John’s Wort as a Treatment for Depression. Cureus.

[31] Zacharia T., Naik P.V., Sada S., Kuniyil J.G., Dwarakanath V.M. (2012). Development and standardization of tinnitus handicap inventory in Kannada. Int. Tinnitus J..

[32] Preljevic V.T., Østhus T.B., Sandvik L., Opjordsmoen S., Nordhus I.H., Os I., Dammen T. (2012). Screening for anxiety and depression in dialysis patients: Comparison of the Hospital Anxiety and Depression Scale and the Beck Depression Inventory. J. Psychosom. Res..

[33] Gu H., Kong W., Yin H., Zheng Y. (2022). Prevalence of sleep impairment in patients with tinnitus: A systematic review and single-arm meta-analysis. Eur. Arch. Otorhinolaryngol..

[34] Brazier J.E., Harper R., Jones N.M., O’Cathain A., Thomas K.J., Usherwood T., Westlake L. (1992). Validating the SF-36 health survey questionnaire: New outcome measure for primary care. BMJ.

[35] Sieghart W. (1994). Pharmacology of benzodiazepine receptors: An update. J. Psychiatry Neurosci..

[36] Johnson R.M., Brummett R., Schleuning A. (1993). Use of alprazolam for relief of tinnitus. A double-blind study. Arch. Otolaryngol. Head Neck Surg..

[37] Jalali M.M., Kousha A., Naghavi S.E., Soleimani R., Banan R. (2009). The effects of alprazolam on tinnitus: A cross-over randomized clinical trial. Med. Sci. Monit..

[38] Jufas N.E., Wood R. (2015). The use of benzodiazepines for tinnitus: Systematic review. J. Laryngol. Otol..

[39] Han J.S., Park J.M., Park S.Y., Vidal J.L., Ashaikh H.K., Kim D.K., Park S.N. (2020). Typewriter tinnitus: An investigative comparison with middle ear myoclonic tinnitus and its long-term therapeutic response to carbamazepine. Auris Nasus Larynx.

[40] Piccirillo J.F., Finnell J., Vlahiotis A., Chole R.A., Spitznagel E. (2007). Relief of idiopathic subjective tinnitus: Is gabapentin effective?. Arch. Otolaryngol. Head Neck Surg..

[41] Bauer C.A., Brozoski T.J. (2006). Effect of gabapentin on the sensation and impact of tinnitus. Laryngoscope.

[42] Farhadi M., Salem M.M., Asghari A., Daneshi A., Mirsalehi M., Mahmoudian S. (2020). Impact of Acamprosate on Chronic Tinnitus: A Randomized-Controlled Trial. Ann. Otol. Rhinol. Laryngol..

[43] Kim D.Y., Kwon O.D., Kim S.G., Shin I.H. (2008). Recognitional study about patients and caregivers’ understanding of clinical trial. J. Korean Cont. Soc..

